# Supplementary material for: Learning curve and surgical outcome of robotic assisted colorectal surgery with ERAS program
Source: Sci Rep. 2022 Nov 29;12:20566. doi: 10.1038/s41598-022-24665-w (PMC9709162; doi:10.1038/s41598-022-24665-w)
Supplement: Supplementary file 2 — Supplementary Information 2. [file 41598_2022_24665_MOESM2_ESM.zip › RAL Raw data-Ñ[▒K/065/OP N_ñw╝╨░O▒Kñσ.pdf]

臺中榮民總醫院
手術室護理記錄

[redacted]

基本資料

- 室別： 開刀房-17室，手術科別： CRS，手術類別： 常規，日期： 2021/03/17
- 進入時間： 1440 ； 麻醉開始時間： 1445 ； Time Out時間： 1549 ； 劃刀時間： 1550 ； 手術結束時間： 1915 送出時間： 1935 ；
- 麻醉方式： GE

  診斷： Malignant tumor of sigmoid colon

  手術名稱： Robot Assisted Colectomy(L)

  手術局部用藥： nil

  術前預防性抗生素：
  - 抗生素名稱一： Veterin 1 gm IVD由麻醉人員給予
  - 時間： 1530
  - 上次簽章: 紀玉娟

  ，追加預防性抗生素：
- 手術醫師： 蔣鋒帆，楊曙榮，林昌霖，劉佳樺
- 麻醉醫師： VS 張思玲
-
  - 刷手護士A ： 高子貽，時間： 1440，刷手護士B ： 章佑禎，時間： 1440，接替刷手一： 李郁萱，時間： 1500，接替刷手二： 黃美雅，時間： 1500
  - 流動護士A ： 何宛臻，時間： 1440，接替流動一： 紀玉娟，時間： 1500，接替流動二： 黃美雅，時間： 1630
- 更多接替及流動護士： 接替刷手三： 紀玉娟，時間： 1700，接替流動三： 李郁萱，時間： 1730

皮膚狀況

A-V Shunt： 無

術前： 完整

記錄者： 上次簽章: 何宛臻

術後： 完整

記錄者： 上次簽章: 李郁萱

手術準備

手術臥式 單一手術臥式

-
  - 手術臥式： 垂頭仰臥
  - 使用附件： 溫毯 38 ℃，頭圈，腳架，側身架，其它： SILICON PAD
  - 皮膚準備： 10%Aq Betadine，Hibiscrub，2% Chlorhexidine
  - 電燒： 使用電燒: 電極片位置： 右大腿，記錄者： 上次簽章: 紀玉娟

止血帶T： 無

植入物

- 植入物名稱/號碼/數量/部位： DA VINCIXI ENDOWRIST STAPLER 45 GREEN RELOAD*1/ COLON
  EEA 33mm/ COLON

，記錄者： 上次簽章: 李郁萱

標本送檢

- 細菌培養 無
- 細胞學 無
- 冰凍切片 無
- 病理標本

  組織名稱一： IMA root

  ,

  組織名稱二： distal cut end

  ,

組織名稱三： S-colon

- 截肢 無

標本交付 標本： 3 袋，標本： 0 盒，標本： 0 瓶，細胞學： 0 管，Culture Tube： 0 管，被交付醫師： CR 楊曙榮，

- 記錄者： 上次簽章: 李郁萱
- 時間： 1900

傷口術後敷料

傷口敷料： 傷口敷料： 無

- 請於圖上標示部位：

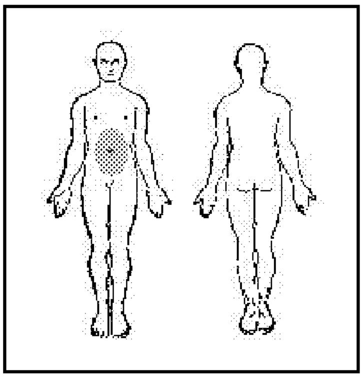

- 傷口敷料： SOFT CLOTH

導管

導管	天數	導管資訊	插入日期	插入時間	更換日期	拔除日期	拔除時間	拔除原因	預計拔除/更換	預計拔除/更換日期
Foley(1)	1	號碼:16 Fr.; 部位:膀胱;	2021/03/17	1525						
Double Lumen CVC(1)	1	號碼:8 Fr; 深度:15cm; 部位:subclavian (左); 象限:LU;	2021/03/17	1510						
A-line (1)	1	號碼:20 G; 部位:左手;	2021/03/17	1500						
Epidural PCA(1)	1	部位:epidural;	2021/03/17	1440						

用物計數

- 手術中出血量： minimal c.c.
- 手術中尿量： 380 c.c.
-
    - 其它： 無

用物計數： 多切口同時縫合

- 1.器械： 正確，2.縫針： 正確，3.刀片： 正確，4.紗布： 正確，5.腹部墊： 未使用，6.紗球： 未使用，7.PEANUTS ： 未使用，8.紗條： 未使用，9.絲帶： 未使用，10.針頭： 未使用，11.Vessel loop： 未使用，12.Boots： 未使用，13.Jelco： 未使用，14.棉條： 未使用，
    - 第一次：
        - 刷手護士 ： 紀玉娟
        - 流動護士： 上次簽章: 李郁萱
        - 時間： 1845

    ,
    - 第二次：
        - 刷手護士 ： 紀玉娟
        - 流動護士： 上次簽章: 李郁萱
        - 時間： 1850

    ,
    - 第三次：
        - 刷手護士 ： 紀玉娟
        - 流動護士： 上次簽章: 李郁萱
        - 時間： 1855

備註

備註： 腹腔鏡 NO.6/XI Davinci scope 30度 NO.2

- 記錄者： 上次簽章: 李郁萱
- 時間： 1935

填寫護理人員 李郁萱

正式摘要

W52 / W52

doctorRank:B
